# Supplementary material for: A Small Molecule Targeting the Intracellular Tyrosine Kinase Domain of ROR1 (KAN0441571C) Induced Significant Apoptosis of Non-Small Cell Lung Cancer (NSCLC) Cells
Source: Pharmaceutics. 2023 Apr 5;15(4):1148. doi: 10.3390/pharmaceutics15041148 (PMC10145660; doi:10.3390/pharmaceutics15041148)
Supplement: Supplementary file 1 [file pharmaceutics-15-01148-s001.zip › pharmaceutics-2251558-supplementary.pdf]

Supplementary Data

Table S1. Relation between ROR1 expression and subgroups of lung cancer patients.

	ROR1 expression *			
**Histology**	**0**	**1**	**2**	**P value**
**Non-Squamous low-differentiated adenocarcinoma (n=157)**	21 (13) **	60 (38)	76 (48)	
**Squamous (n=106)**	46 (43)	32 (30)	28 (26)	0,0001
**Neuroendocrine (mainly carcinoids) (n=24)**	19 (80)	2 (8)	3 (12)	

* ROR1 expression was scored as: 0: negative; 1: weak; 2: strong
** Figures within brackets indicate percent

Table S2. ROR1 expression by pathological stage (7th TNM).

Pathological stage	ROR1 expression intensity *		
	0	1	2
**IA n= (118)**	28 (24) **	45 (38)	45 (38)
**IB n= (89)**	21 (24)	27 (30)	41 (46)
**II n= (40)**	14 (35)	17 (42)	9 (22)
**III-IV (n=9)**	4 (25)	3 (19)	9 (56)

* ROR1 expression scored as: 0: negative; 1: weak; 2: strong. ** Figures within brackets indicate percent.

Table S3. Characteristics of the ROR1 positive lung cancer cell lines.

Cell lines	Histology	Doubling time (hours)	EGFR mutation	K-RAS mutation	P53 mutation
**NCI-H1975**	adenocarcinoma	53	Mutated L858R/T790M	WT *	+
**NCI-H23**	adenocarcinoma	33	WT	+	+
**NCI-HCC827**	adenocarcinoma	44	Mutated E746-A750 deletion (Del19)	WT	N/A **
**A549**	adenocarcinoma	23.8	WT	+	WT
**NCI-H1299**	squamous cell carcinoma	23.1	WT	WT	+

* Wild type, ** not available.

Table S4. Clinical characteristics of adenocarcinoma NSCLC patients (n=139) in relation to ROR1 expression.

Patients characteristics	ROR1 $^{-}$ patients (n=16)	ROR1 $^{+}$ patients (n=123)	P value
**Age (y), Median**	69 (56-72)	65 (56-72)	0.5
**Gender**			
Male	4 (3) *	59 (42)	0.07
Female	12 (9)	64 (46)	
**Smoking status**			
Current	9 (6)	54 (39)	
Former	5 (4)	38 (27)	0.6
Never	1 (1)	18 (13)	
Missing	1 (1)	13 (9)	
**Type of surgery**			
Lobectomy	12 (9)	98 (70)	0.6
Pulmectomy	4 (3)	26 (18)	
**P Stage (7th TNM)**			
IA	10 (7)	62 (45)	
IB	2 (1)	38 (27)	0.4
II	3 (2)	17 (12)	
III-IV	1 (1)	6 (4)	
**Histologic Grading**			
G1	3 (2)	46 (35)	
G2	6 (5)	35 (26)	0.3
G3	6 (5)	36 (27)	
**P53 Expression**			
Negative	7 (5)	88 (63)	0.03
Positive	9 (6)	35 (25)	

* Figures within brackets indicate percent.

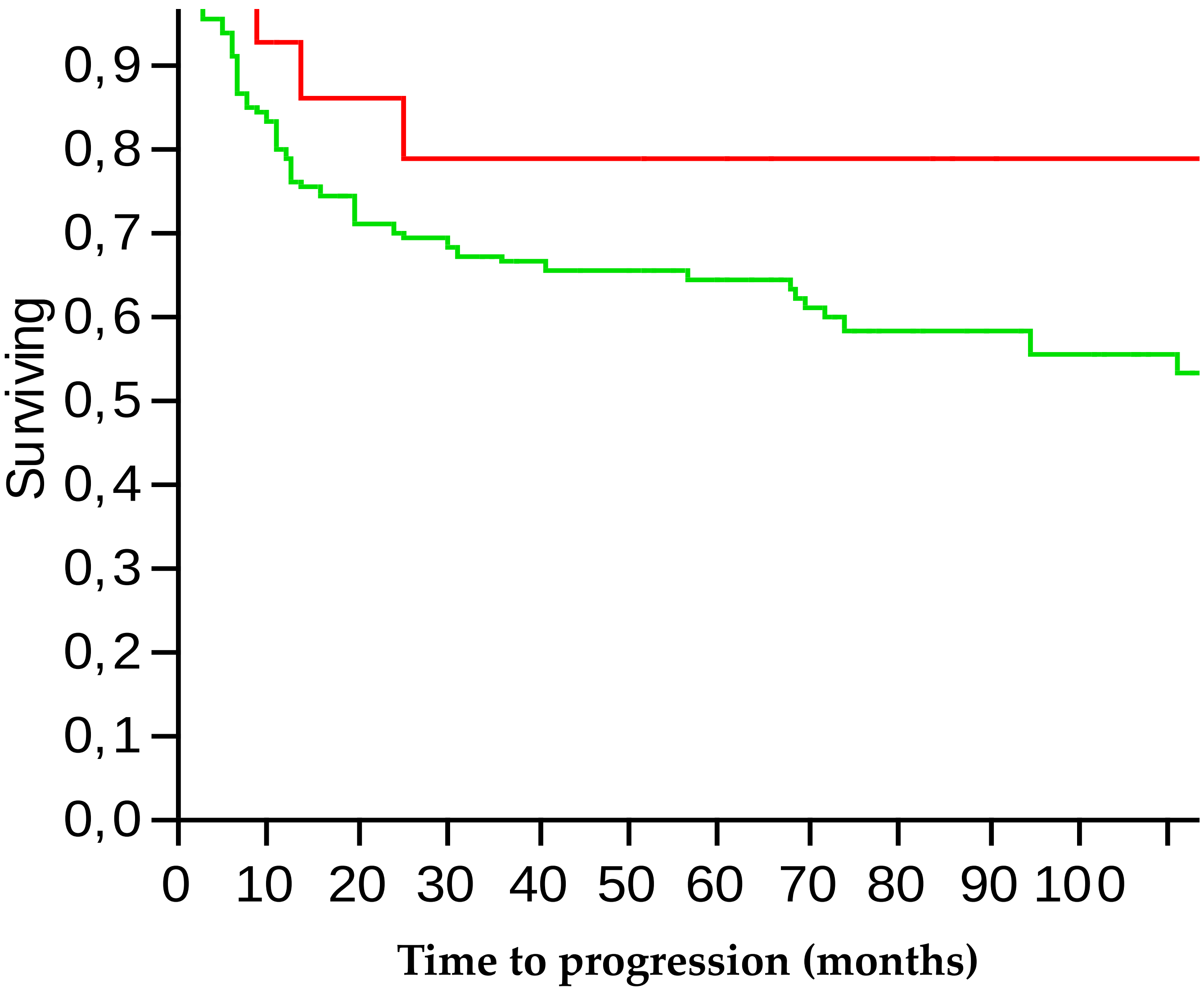

Fig S1: Time to progression (TTP) in non-squamous NSCLC patients with no ROR1 expression (red line) (n=16) compared to those positive for ROR1 (green line) (n=123) (p=0.09).

Fig S2. Immunofluorescent staining of untreated lung cancer cell lines using anti-ROR1 antibodies (20X). Lung cancer cells were cultured on sterile 8 wells glass slide for 24 h, fixed, blocked in PBS containing BSA and then incubated with an anti-ROR1 antibody for 24 h at 4°C and finally treated with Alexa Flour 488-conjugated goat anti-mouse IgG for 1 h. After washing, the cell nuclei were stained with mounting media containing DAPI. A Zeiss Axioplan2 fluorescence microscope was used.

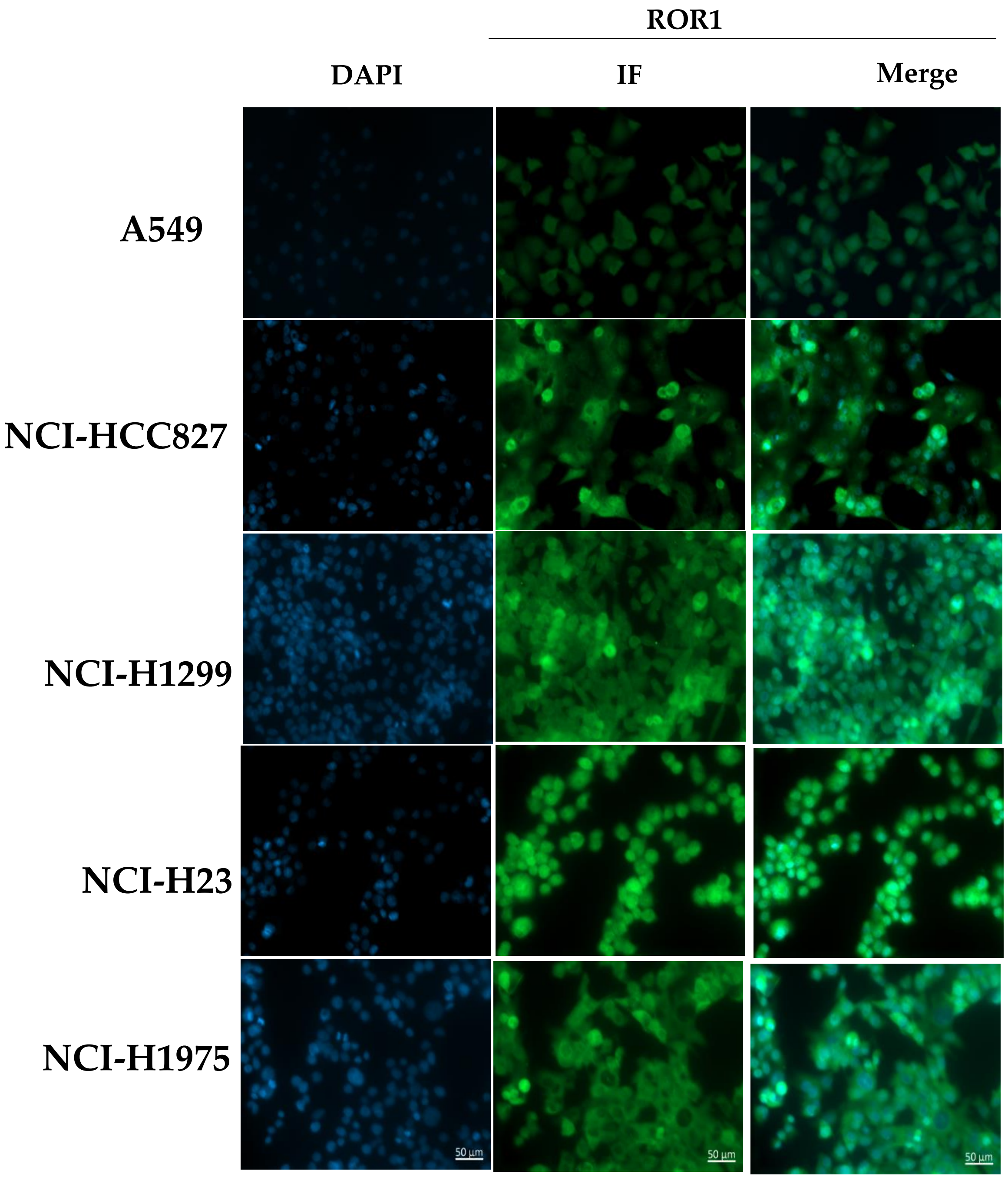

Fig S3. Migration of lung cancer cell lines (NCI-H1299 and NCI-H1975) in the wound scratch healing assay. Migration of lung cancer cell lines was inhibited after treatment with 50-1000 nM of KAN0441571C for 0, 24 and 72 h.

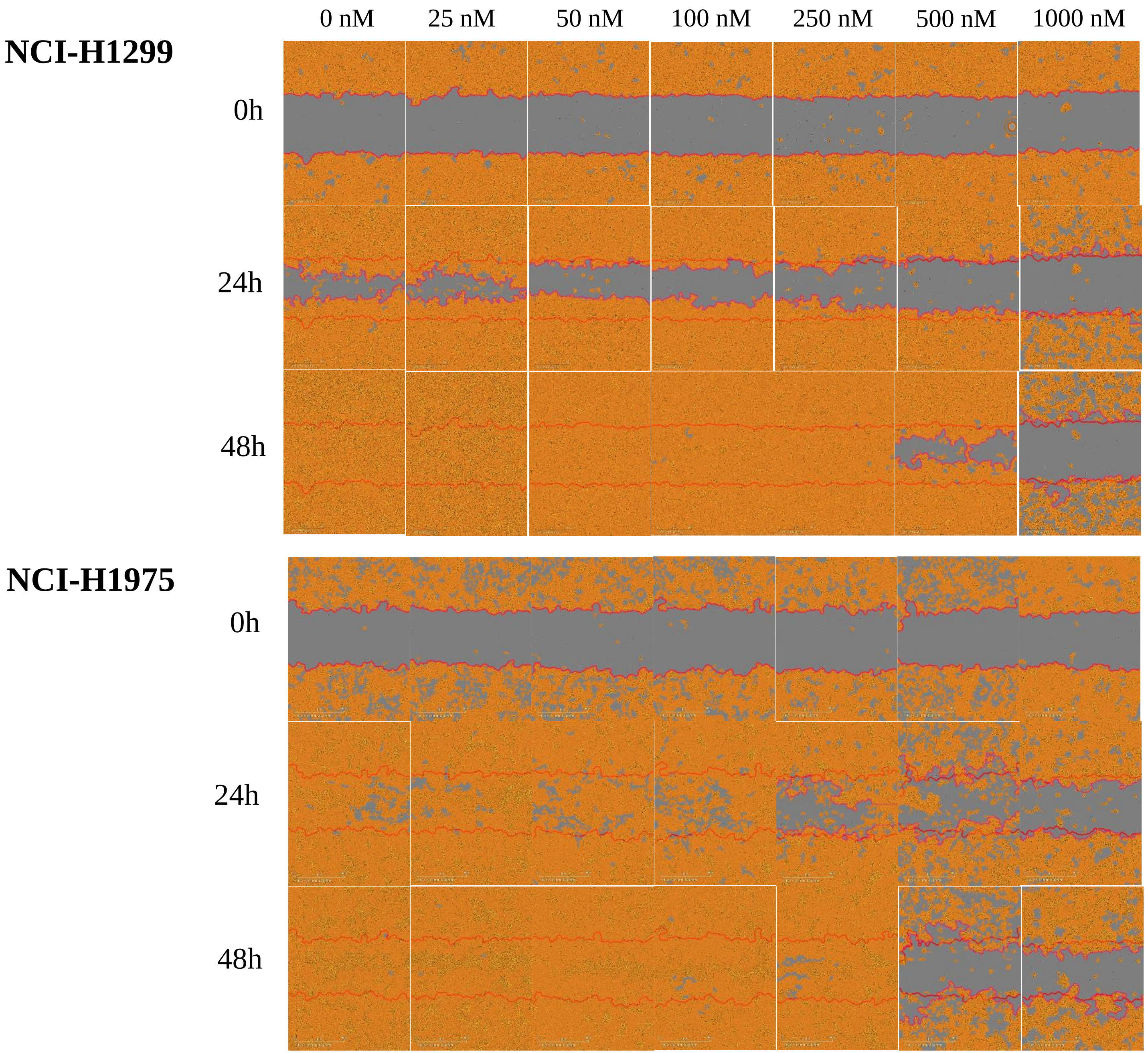

Figure S4. Cell cycle profiles of A549 lung cancer cells treated with KAN0441571C. KAN0441571C induced reduction of S phase cells and accumulation in the G2 phase with a simultaneous increased cell death. Representative histograms (A) and quantifications (B) of 3 independent experiments.

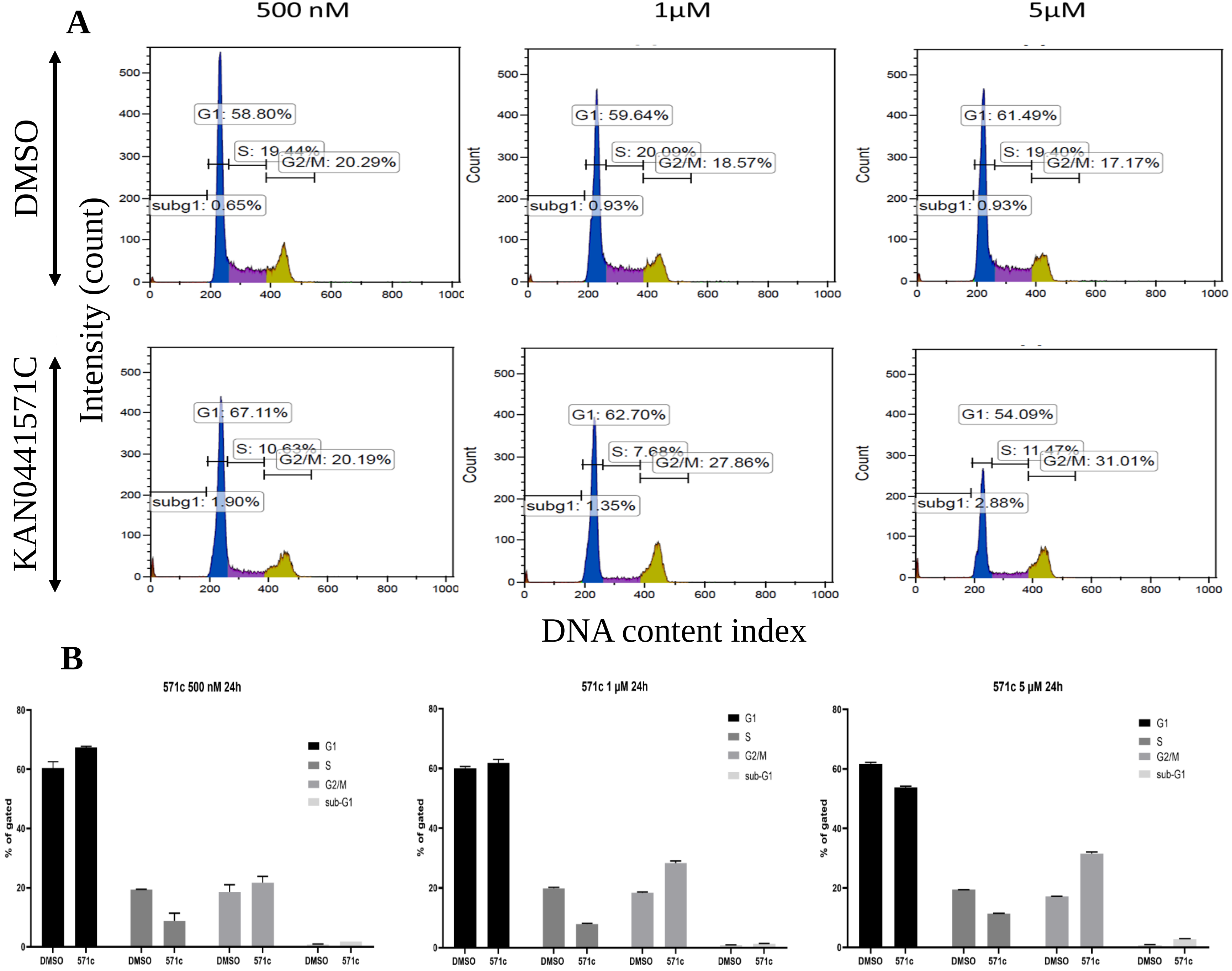

Figure S5. Effect of KAN0441571C on ROR1 associated signaling molecules (Western blot) (4 h incubation) in the lung cancer cell line NCI-H23. ROR1, EGFR, SRC, AKT, PI3K110δ, mTOR, and CREB were dephosphorylated. One of three representative experiments is shown.

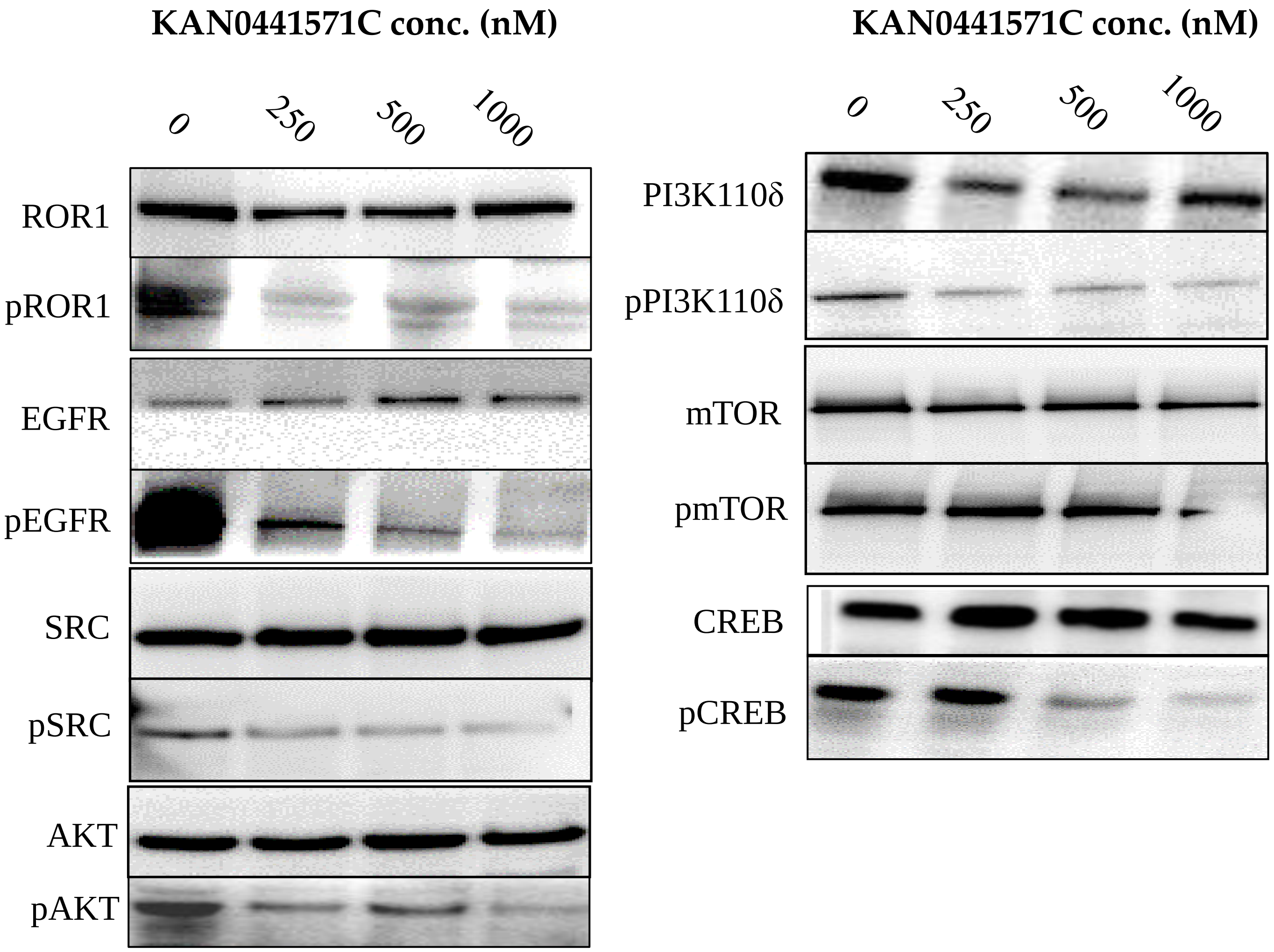

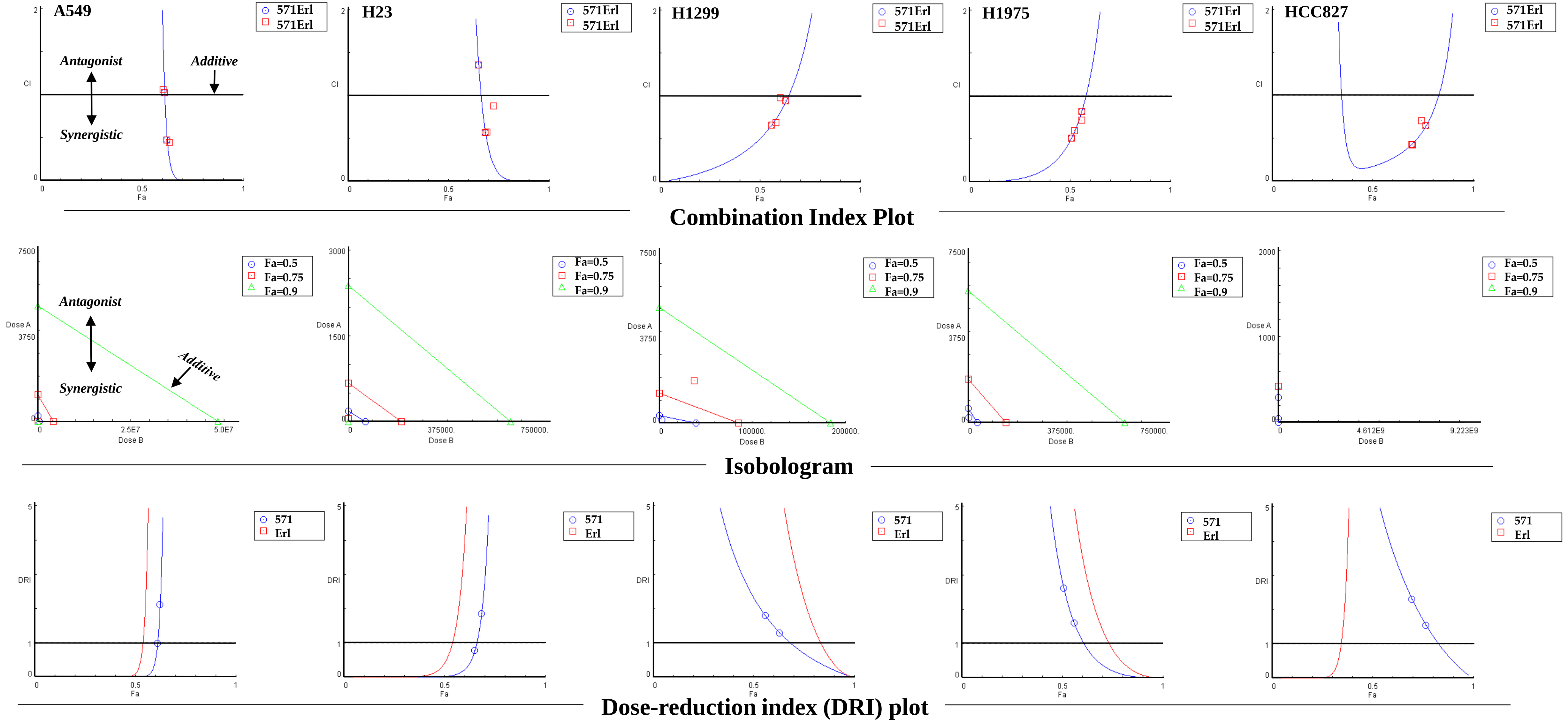

Figure S6A. Apoptosis (Annexin V/PI) in 5 lung cancer cell lines incubated with a combination of KAN0441571C (250 nM) with erlotinib (5000-10000 nM). For each combination, data are shown as combination index plot (CI), isobologram and dose reduction index (DRI). Combination Index (CI) Plot (Fa-CI plot): Values across Fa in lung cancer cells, where Fa is the % of cell death. CI <1 synergistic effect; CI=1 additive effect; CI >1 antagonistic effect. (Chou/Talalay) method).

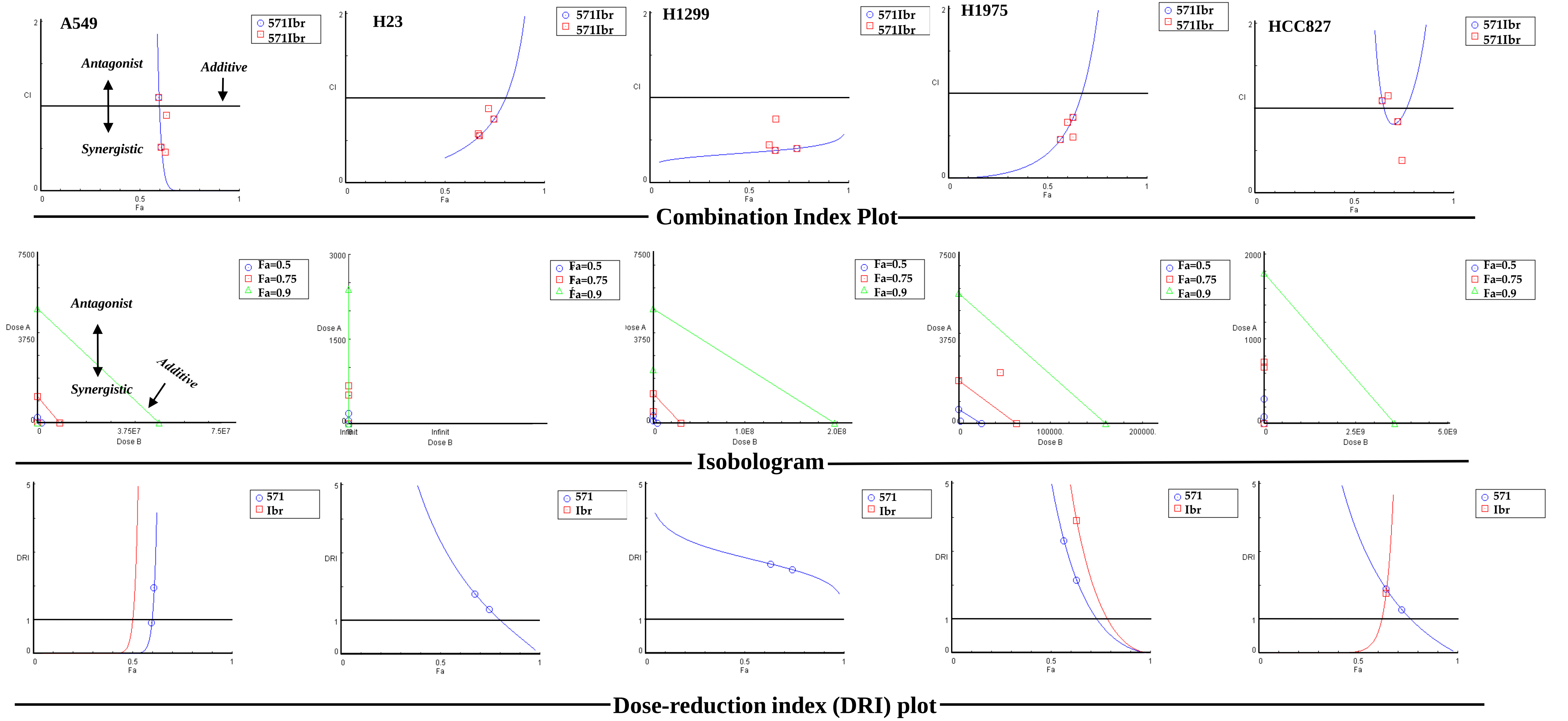

Figure S6B. Apoptosis (Annexin V/PI) in 5 lung cancer cell lines incubated with a combination of KAN0441571C (250nM) with ibrutinib (5000-10000nM). For each combination, data are shown as combination index plot (CI), isobologram and does reduction index (DRI). Combination Index (CI) plot (Fa-CI-plot): Values across Fa in lung cancer cell lines, where Fa is the % of cell death. CI<1 synergistic effect; CI=1 additive effect; CI > 1 antagonistic effect. (Chou/Talalay method).
